# Bioenergetics of the Calf Muscle in Friedreich Ataxia Patients Measured by 31P-MRS Before and After Treatment with Recombinant Human Erythropoietin

**DOI:** 10.1371/journal.pone.0069229

**Published:** 2013-07-29

**Authors:** Wolfgang Nachbauer, Sylvia Boesch, Rainer Schneider, Andreas Eigentler, Julia Wanschitz, Werner Poewe, Michael Schocke

**Affiliations:** 1 Department of Neurology, Medical University Innsbruck, Innsbruck, Austria; 2 Department of Biochemistry, Leopold-Franzens-University Innsbruck, Innsbruck, Austria; 3 Department of Radiology, Medical University Innsbruck, Innsbruck, Austria; National Institute for Medical Research, Medical Research Council, London, United Kingdom

## Abstract

Friedreich ataxia (FRDA) is caused by a GAA repeat expansion in the FXN gene leading to reduced expression of the mitochondrial protein frataxin. Recombinant human erythropoietin (rhuEPO) is suggested to increase frataxin levels, alter mitochondrial function and improve clinical scores in FRDA patients. Aim of the present pilot study was to investigate mitochondrial metabolism of skeletal muscle tissue in FRDA patients and examine effects of rhuEPO administration by phosphorus 31 magnetic resonance spectroscopy (31P MRS). Seven genetically confirmed FRDA patients underwent 31P MRS of the calf muscles using a rest-exercise-recovery protocol before and after receiving 3000 IU of rhuEPO for eight weeks. FRDA patients showed more rapid phosphocreatine (PCr) depletion and increased accumulation of inorganic phosphate (Pi) during incremental exercise as compared to controls. After maximal exhaustive exercise prolonged regeneration of PCR and slowed decline in Pi can be seen in FRDA. PCr regeneration as hallmark of mitochondrial ATP production revealed correlation to activity of complex II/III of the respiratory chain and to demographic values. PCr and Pi kinetics were not influenced by rhuEPO administration. Our results confirm mitochondrial dysfunction and exercise intolerance due to impaired oxidative phosphorylation in skeletal muscle tissue of FRDA patients. MRS did not show improved mitochondrial bioenergetics after eight weeks of rhuEPO exposition in skeletal muscle tissue of FRDA patients.

**Trial Registration:**

*EU Clinical Trials Register* 2008-000040-13

## Introduction

Friedreich Ataxia (FRDA) is the most common inherited ataxia among Caucasian populations. Clinically, FRDA is determined by progressive ataxia of gait and extremities, dysarthria and areflexia [1]. Muscle weakness is evident particularly in the lower limbs [2], [3], though signs of weakness or fatigue may be masked by more prominent features of ataxia. FRDA is a caused by a GAA trinucleotid expansion in intron 1 of the frataxin gene [4] leading to reduced expression of the mitochondrial protein frataxin. Frataxin is critically involved in iron sulfur cluster assembly, respiratory chain activity and iron homeostasis within mitochondria [5], [6]. The pathological cascade in FRDA is not solely restricted to the nervous system. Besides, muscle tissue especially cardiac muscle but also skeletal muscle show decreased frataxin expression and impairment of the respiratory chain [7], [8]. Reduced skeletal muscle adenosine triphosphate (ATP) production and mitochondrial dysfunction could be demonstrated earlier by magnetic resonance spectroscopy (MRS) [9], [10]. Furthermore MRS was used for monitoring therapeutics in FRDA [11]. Recombinant human Erythropoietin (rhuEPO) has shown to up-regulate frataxin levels in-vitro and in-vivo [12], [13], [14]. Moreover, decrease in oxidative stress [15] and changes in mitochondrial function [16] have been reported after rhuEPO application in FRDA.

Phosphorus 31 magnetic resonance spectroscopy (31P MRS) offers a non invasive investigation of human skeletal muscle bioenergetics by monitoring relative and absolute changes of phosphocreatine (PCr), inorganic phosphate (Pi) and adenosine triphosphate (ATP) during incremental exercise and recovery [17]. ATP production in skeletal muscle tissue relies on three main sources. In absence of sufficient oxygen supply ATP synthesis is dependent on PCr and glycolysis, whereas mitochondrial oxidative phosphorylation (OXPHOS) requires oxygen and substrates such as pyruvate or fatty acids [18]. During exercise PCr decreases continuously until oxygen supply is increased sufficiently and adenosine triphosphate (ATP) can be provided for rephosphorylation of PCr [19]. Particularly PCr regeneration after exercise is an indicator for mitochondrial function in skeletal muscle [20]. Aim of the present study was to investigate mitochondrial function of skeletal muscle tissue in FRDA patients and to examine effects of rhuEPO application.

## Patients and Methods

The protocol for this trial and supporting CONSORT checklist are available as supporting information; see Checklist S1 and Protocol S1.

### Ethics Statement

The study was approved by the local ethics committee of the Medical University of Innsbruck (approval number: UN3152_LEK) and is conducted according to the principles expressed in the Declaration of Helsinki. The trial is registered in the EudraCT database (number: 2008-000040-13). All participants gave written informed consent.

### Patients

**Figure 1:**
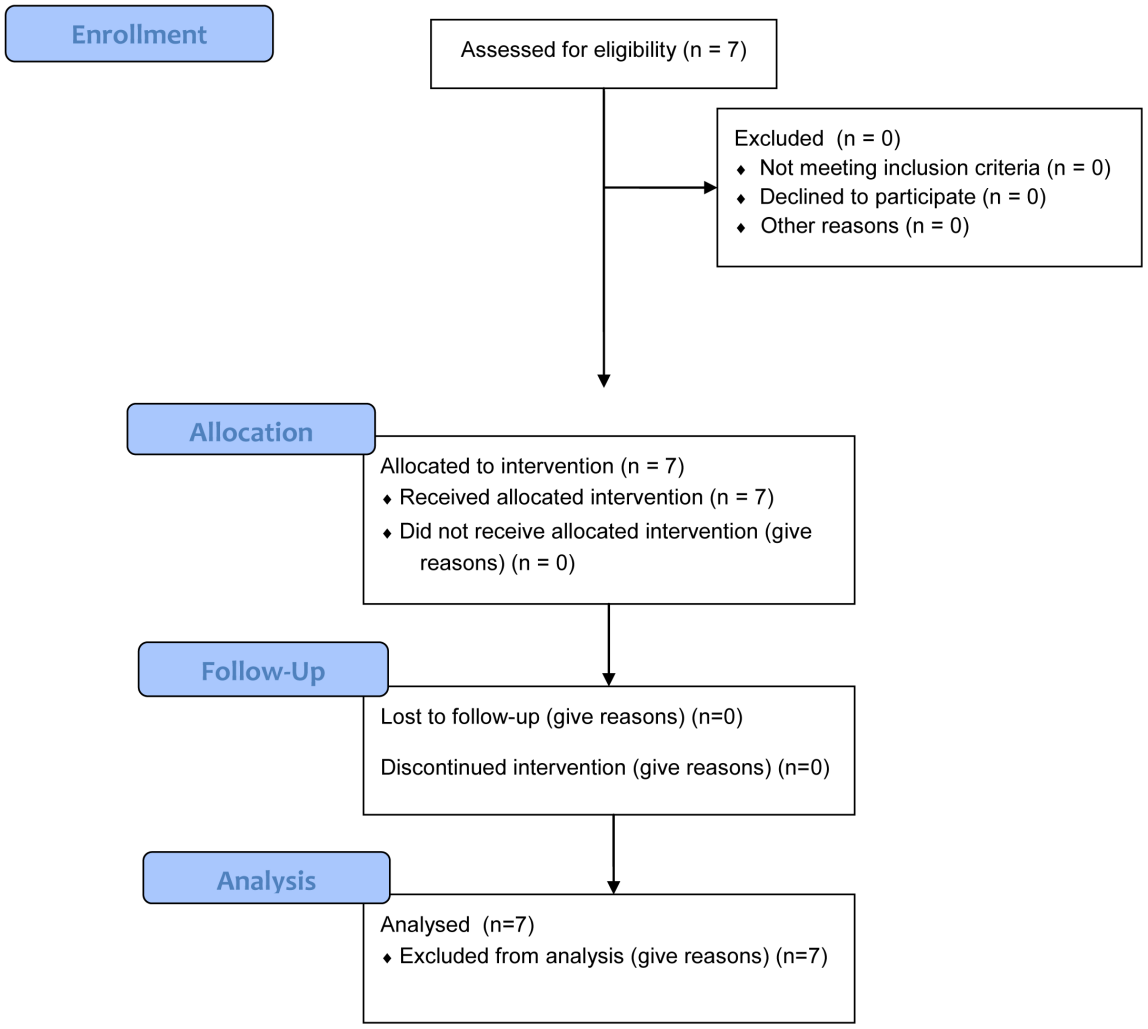
CONSORT Flow Diagram.

Seven patients (1 female, 6 male) were included in this open label pilot study (compare figure 1). All patients were recruited from the ataxia outpatient clinic of the Department of Neurology (Medical University Innsbruck). Pre-recruitment from the investigator site’s own patient pool started in October 2008. Screening visits to check for inclusion and exclusion criteria were carried out in February and March 2009 after final trial registration. Administration of the investigational medicinal product and study related procedures were conducted in the time period from March to June 2009. Changes to initial trial protocol were not performed throughout the study. All patients were older than 18 years (40±14 years), had genetically confirmed FRDA with a mean GAA length of the shorter allele of 584±337 repeats and displayed a disease duration of 18±8 years on average (*compare* *table 1*). As an age- and sex- matched control group for MRS bioenergetics 8 healthy adults (2 female, 6 male) without evidence of neurological disease were included. Exclusion criteria for participation were a hematocrit greater than 50% or thrombocytosis at screening visit. Furthermore, patients with cancer, chronic inflammatory diseases, unstable diabetes mellitus (HbA1c>8%), chronic liver disease, alcohol abuse, epilepsy, heart insufficiency (NYHA>2), previous thrombo-embolic events, anti-coagulation, pregnancy or breast feeding, iron, vitamin B12 and/or folic acid deficiency, cardiovascular diseases, psychiatric disorders, hypersensitivity to rhuEPO or patients participating in different clinical trials were excluded from the study. Primary outcome was to define PCr and Pi time course during exercise and recovery in skeletal muscle of FRDA patients. Secondary outcome was to investigate mitochondrial function in response to rhuEPO treatment. No changes to eligibility criteria or outcome measures were made after trial commencement.

**Table 1:** Demographics and concentration of metabolites.

	FRDA baseline		FRDA rhuEPO		Controls		baseline-rhuEPO	FRDA-Controls
	mean	SD	mean	SD	mean	SD	p value	p value
Age	40.00	14.01			40.06	13.26	n.a.	0.933
SARA	23.07	8.20	20.34	7.86	0.00	0.00	0.003	0.000
Repeats	584.29	337.53					n.a.	n.a.
Duration	18.14	8.31					n.a.	n.a.
**Quantification of metabolite (mmol/l)**								
**Resting state**								
PCr	37.52	3.94	37.54	4.51	37.08	1.60	0.861	0.714
Pi	3.42	0.78	3.57	0.86	2.85	0.44	0.463	**0.010**
pH	7.04	0.03	7.05	0.03	7.05	0.04	0.959	0.449
**Exercise abruption**								
PCr	22.89	8.36	20.82	5.42	18.51	3.03	0.445	0.205
Pi	11.65	4.43	12.94	4.50	12.97	1.58	0.721	0.837
pH	6.99	0.15	6.97	0.12	6.95	0.08	0.972	0.948
**End of regeneration**								
PCr	38.12	4.26	35.03	3.32	38.61	2.52	0.064	0.619
Pi	2.32	0.89	2.59	1.02	1.62	0.49	0.695	**0.020**
pH	7.07	0.20	6.93	0.10	7.01	0.03	**0.041**	0.475

Demographical and clinical values of FRDA patients before (*FRDA baseline*) and after stimulation (*FRDA rhuEPO*) with rhuEPO shown as comparison to healthy control subjects (*Controls*). Absolute concentrations of metabolites (PCr, Pi) in resting state, at exercise abruption and at the end of regeneration are given as mmol/l by using ATP as an internal standard. Intracellular pH was calculated from the chemical shift of Pi.

*Values are given as mean and standard deviation (SD). Abbreviations: SARA (scale for the assessment and rating of ataxia), Repeats (GAA repeat of the shorter allele is given), Duration (disease duration), PCr (phosphocreatine), Pi (inorganic phosphate), FRDA (Friedreich ataxia), rhuEPO (recombinant human erythropoietin*).

### Study Design

FRDA patients received 3,000 international units (IU) rhuEPO (Roche, Switzerland) thrice weekly over a study period of 8 weeks. 31P MRS was carried out before and after rhuEPO treatment. Biochemical measurements of respiratory chain activity and frataxin levels were performed in skeletal muscle tissue pre- and post rhuEPO treatment. The “Scale for the Assessment and Rating of Ataxia” (SARA, [21], [22] was used for monitoring clinical changes. Safety was assessed by red blood cell count and blood pressure in two weekly intervals.

### 31P MRS Protocol

To avoid confounding effects all FRDA patients underwent 31P MRS before muscle biopsy. MRS was carried out performing one exercise cycle each for both legs. The exercise bench consisted of a pedal ergometer that was connected to a piston and an air-pressure-powered cylinder [23], [24], [25], [26]. Stable power output during one increment was assured by a regulation software that recorded continuously force, distance and frequency during pedal movement [27]. The protocol consisted of measurements at rest and during exercise increments at 1, 2, 4 and 6 W. MRS was carried out on a 1.5 Tesla whole body MR scanner (Magnetom Magnetom Avanto, Siemens Erlangen, Germany), using a circular polarized double resonator surface coil that permits the receipt of 1H resonances at 63.5 MHz and 31P resonances at 25.8 MHz. The diameter of the transmitter coil was 21 cm and the diameter of the receiver coil 14 cm. A free induction decay sequence with a repetition time of 1000 ms, an echo time of 0.13 ms, a flip angle of 90°, 10 averages and an acquisition time of 10 s was applied. The nuclear Overhauser enhancement (NOE) was applied to all MRS measurements [28], [29]. The signal was received from the calf that was fixed on the double resonator coil.

### 31P MRS Data Analyses

Spectroscopic measures were processed using the commercial software package as provided by the manufacturer (Siemens Erlangen, Germany). The peak areas of PCr, Pi and ATP were fitted in the frequency domain. Additionally, the position of the peaks of PCr and Pi were determined. Absolute concentrations of PCr and Pi were calculated using ATP as an internal standard by assuming an ATP concentration of 8.2 mmol/l [30]. Intracellular pH was calculated from the chemical shift of Pi based on following equation [31]:
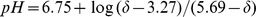
δ: chemical shift of the Pi peak in parts per million (ppm) relative to PCr.

The time constants τ and Δ_ss_ values, meaning the difference between baseline and steady-state levels, were calculated for PCr and Pi for each incremental workload by using a non-linear regression analysis in R for windows (The R Foundation for Statistical Computing, GNU General Public License, Boston, USA) as previously reported [32]. For that purpose, the last integral of the preceding increment or rest phase was considered as baseline integral for the following increment. The time constants τ and Δ_ss_ values were calculated by using the equation [23], [27], [33]:
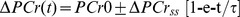


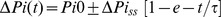
PCr0/Pi0: baseline value

ΔPCr(t)/ΔPi(t): PCr/Pi value at the time (t)

ΔPCr_ss_Δ/Pi_ss_: difference between baseline value and the estimated steady-state level

The coherence between the mono-exponential model and the data of each subject is described by a coefficient of determination r^2^ [23], [27], [33]:
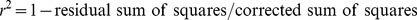



A coefficient r^2^ above 0.4 was considered as an acceptable fit, whereas a coefficient r^2^ below 0.4 indicated a weak agreement with the mono-exponential model and were not used for calculation of time constants.

### Biochemical Analyses

Biochemical analyses were performed from skeletal muscle specimens obtained by open muscle biopsy after local anaesthesia. Skeletal muscle biopsy was performed after MRS at the right gastrocnemius muscle at baseline and at the left gastrocnemius muscle after rhuEPO treatment, respectively. Skeletal muscle tissue was immediately snap-frozen after biopsy in liquid nitrogen and stored at −70 degrees Celsius until analysis. Biochemical analyses included NADH-CoQ-Oxidoreductase (Complex I), Succinat/CytochromC-Oxidoreductase (Complex II/III), CytochromC-Oxidase (Complex IV) and Citratsynthase (CS). Frataxin levels in skeletal muscle tissue were quantified pre- and post rhuEPO treatment by electrochemiluminescence assay as previously reported [7], [34].

### Statistical Analysis

Statistical analyses were carried out using SPSS 17.0 statistical software (Chicago, Illinois). Continuous data are shown as mean values with standard deviation. Measurements were taken from both legs in each patient and control subject and analysed separately. Non parametric tests were used throughout the analyses due to a skewed distribution assessed by Shapiro-Wilk test of most obtained data. Wilcoxon test was applied for assessing differences in time constants and absolute concentrations in dependence to rhuEPO exposure. For comparison to the control subjects Mann-Whitney-U test was used. ANOVA for repeated measurements was applied for evaluating the time course of PCr, Pi and pH. Correlations of quantitative variables were assessed by Pearson bivariate correlation analyses. Two-tailed p values <0.05 were considered as statistically significant.

## Results

The MRS protocol consisted of measurements during resting state, incremental exercise (plantar flexion) and recovery after maximal exhaustive exercise. Analyses in 7 FRDA patients (corresponding to 14 leg analyses) and 8 age-matched controls (corresponding to 16 leg analyses) were included in the present study. Quantification of metabolites at rest revealed elevated absolute levels of Pi (p = 0.010) in FRDA patients, while PCr concentrations were similar (p = 0.714) in patients and control subjects. FRDA patients showed pH values of 7.04±0.03 in resting skeletal muscle, which is comparable to healthy controls (7.05±0.04; p = 0.449). *Compare* *Table 1* *for absolute concentrations of metabolites.*

At exercise abruption absolute concentrations of PCr and Pi were similar in FRDA patients and healthy controls. Pi increased in both groups (FRDA: 11.65±4.43, controls: 12.97±1.58; p = 0.837), while PCr levels declined in parallel (FRDA: 22.89±8.36, controls: 18.51±3.03; p = 0.205). FRDA patients, however, showed earlier exercise abruption in terms of successfully managed increments as compared to controls. Whereas all healthy control subjects completed increment 1 and 2 with both legs (16/16), 6 out of 14 FRDA legs showed progressive PCr breakdown within increment 1 and 4 legs within increment 2 respectively. Terminal PCr breakdown does not lead to steady state conditions and was therefore not considered for calculation of time constants. At exercise abruption, however, controls (6.95±0.08) and patients (6.99±0.15; p = 0.948) showed similar pH-values independent from individually performed exercise increments, which allows for comparison of time constants in the recovery phase [18], [35]. *Compare* *table 1* *for details.*

### PCr and Pi Time Course during Incremental Exercise

During repetitive plantar flexion subsequent decrease in PCr levels was obtained in FRDA patients (p<0.001). FRDA patients showed lower steady state levels of PCr compared to the control group (p = 0.002) as indicated by increased ΔPCr_ss_ values (23.94±12.89 vs. 13.14±5.88 in controls) in increment 1. Time constant τ was exceeded in the FRDA group, although these values did not reach statistical significance (p = 0.298). After rhuEPO exposure PCr time constants τ (p = 0.249) and ΔPCr_ss_ (p = 0.917) did not differ to baseline values (see table 2).

**Table 2:** Time constants of phosphocreatine and inorganic phosphate.

	FRDA baseline		FRDA rhuEPO		Controls		baseline-rhuEPO	FRDA-Controls
	mean	SD	mean	SD	mean	SD	p value	p value
**Exercise**								
ΔPCr_ss_	23.94	12.89	29.22	20.98	13.14	5.88	0.917	**0.002**
PCr τ	33.51	13.21	28.26	18.57	27.95	12.98	0.249	0.298
PCr r2	0.88	0.09	0.79	0.26	0.70	0.18	0.063	**0.007**
ΔPi_ss_	258.65	247.43	213.50	168.51	88.51	44.10	0.484	**0.011**
Pi tau	71.52	69.18	66.22	56.19	24.94	7.34	0.401	**0.005**
Pi r2	0.92	0.08	0.74	0.28	0.70	0.18	0.063	**0.001**
**Recovery**								
ΔPCr_ss_	74.47	50.00	75.60	56.48	105.81	24.31	0.937	0.054
PCr τ	52.80	20.74	55.21	18.00	32.70	5.25	0.937	**0.002**
PCr r2	0.94	0.09	0.91	0.15	0.97	0.02	0.398	0.767
ΔPi_ss_	80.23	11.60	73.55	20.90	89.71	4.14	0.859	**0.002**
Pi τ	62.09	15.62	65.58	40.77	28.20	9.37	0.859	**0.000**
Pi r2	0.86	0.07	0.79	0.19	0.91	0.80	0.477	**0.019**

PCr and Pi time constants (τ) and steady state levels (Δ_ss_) for FRDA patients before (*FRDA baseline*) and after (*FRDA rhuEPO*) erythropoietin stimulation are shown as comparison to the corresponding control group. Values are given as mean and standard deviation (SD) sub-classified for incremental exercise and recovery period. For graphical illustration compare also *figure 3*.

*Abbreviations: PCr (phosphocreatine), Pi (inorganic phosphate), FRDA (Friedreich ataxia), rhuEPO (recombinant human erythropoietin).*

**Figure 2:**
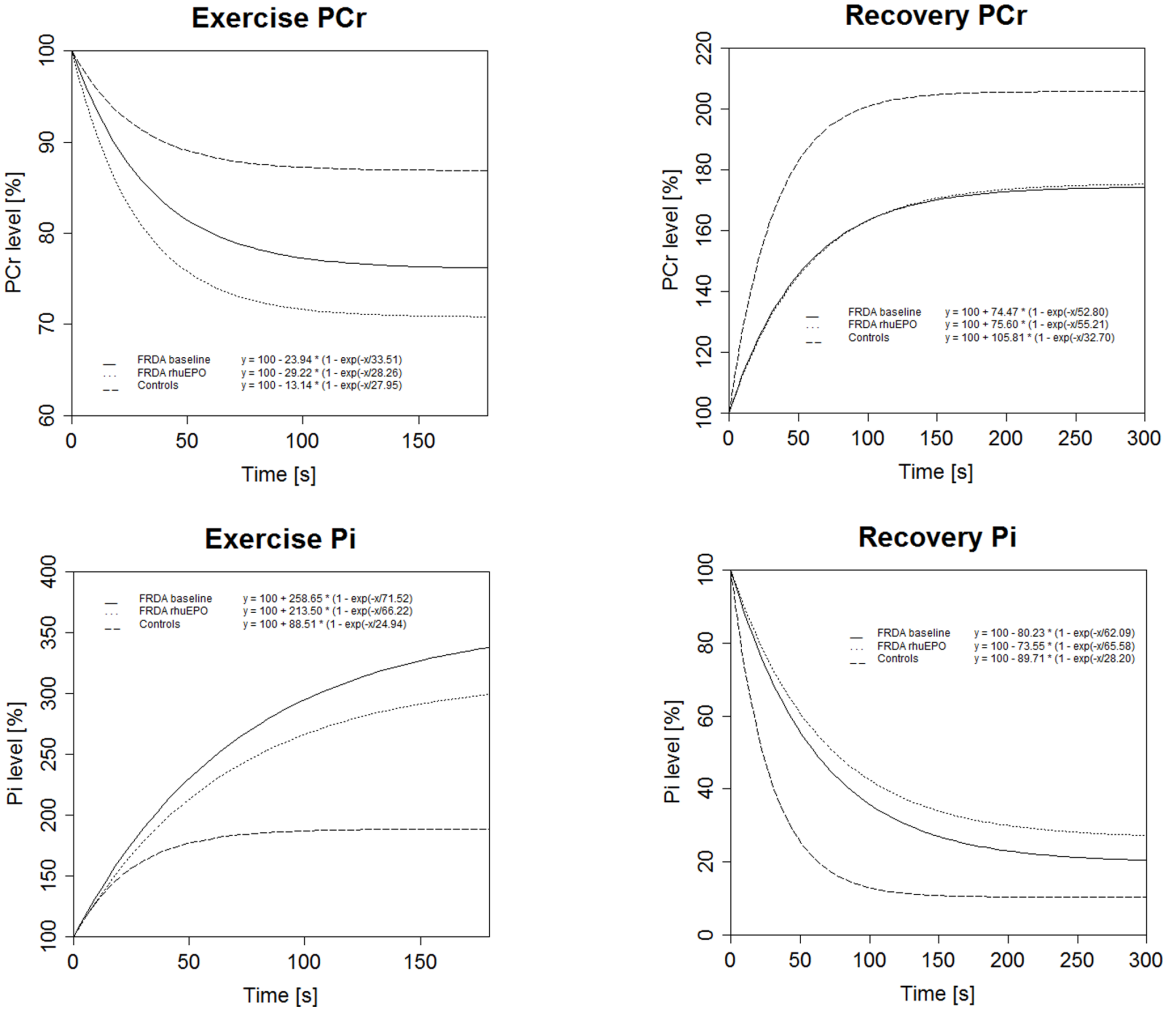
Phosphocreatine and inorganic phosphate kinetics. Time course of phosphocreatine (PCr) and inorganic phosphate (Pi) are shown during exercise in increment 1 and recovery period after maximal exhaustive exercise. Curves are shown as a comparison of healthy controls (*dashed curves*) to treatment naïve FRDA patients at baseline (*solid curves*) and FRDA patients after rhuEPO administration (*dotted curves*). Values are given as a function of time (x) in percentage of changes in PCr and Pi from baseline of the respective increment (100%) and are based on the asymptotic exponential regression model 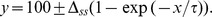

In accordance with PCr depletion Pi increase (p<0.001) could be detected in FRDA patients during incremental exercising. Pi Time constants τ (p = 0.011) and ΔPi_ss_ were exceeded in FRDA, accounting for increased and prolonged accumulation of Pi in relation to controls. Pi increase in FRDA was obtained irrespective of rhuEPO administration (τ: p = 0.401; ΔPi_ss_ p = 0.484). *For detailed values compare* *figure 2* *and* *table 2*.

### PCr and Pi Time Course during Recovery Period

**Figure 3:**
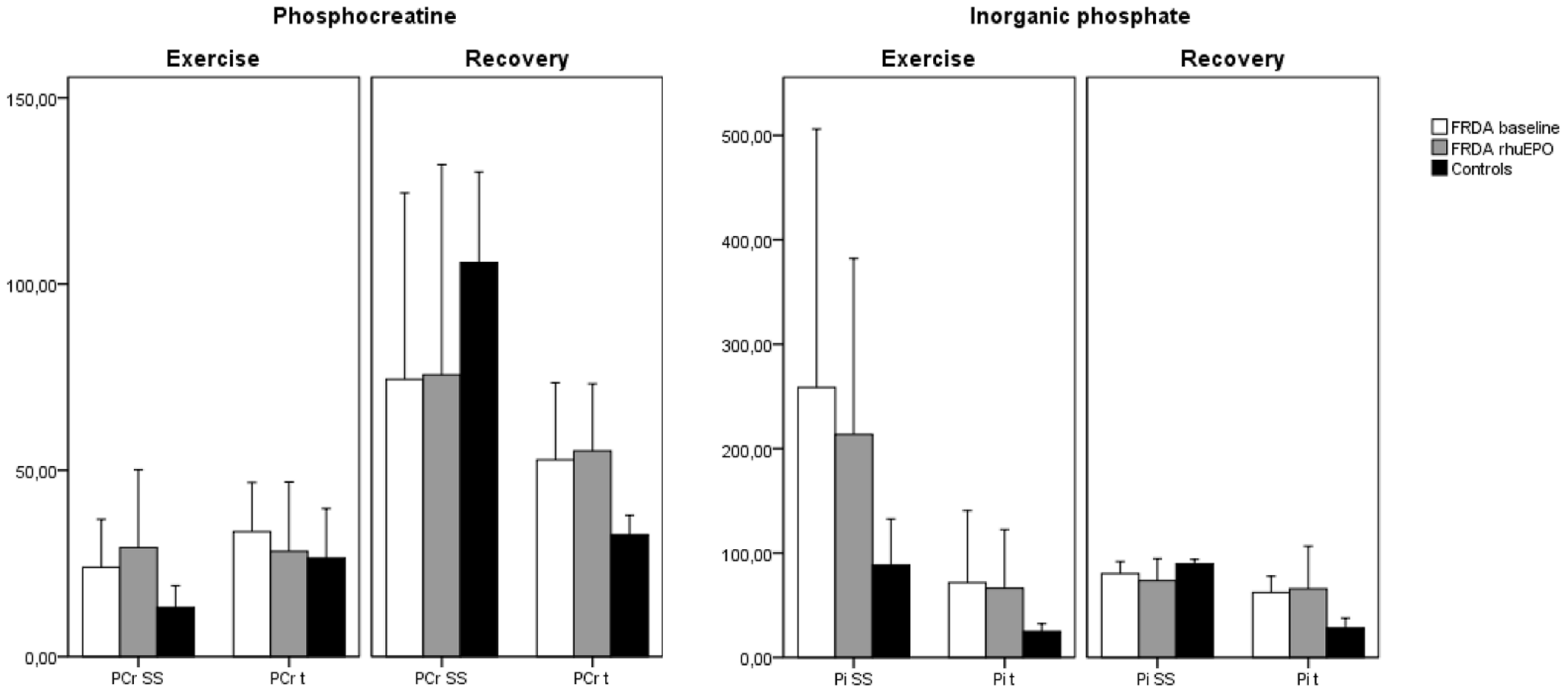
Time constants of phosphocreatine and inorganic phosphate. PCr and Pi time constants (t) and steady state levels (SS) are given during exercise and recovery as a comparison of FRDA patients before (*FRDA baseline*) and after (*FRDA rhuEPO*) erythropoietin stimulation to healthy control subjects. Values are shown as mean and standard deviation. For absolute values compare *table 2*.

During the recovery period regeneration of PCr (p<0.001), progressive loss of Pi (p<0.001) and changes in pH (p = 0.108) were detected. Time for reaching steady state conditions of PCr was prolonged in FRDA as shown by increased PCr time constant τ (p = 0.002). Moreover, decreased steady state of PCr resynthesis (ΔPCr_ss,_ p = 0.054) could be detected in FRDA as compared to control subjects. Pi decline in FRDA patients showed both increased time constant τ (p<0.001), as well as increased steady state conditions of Pi (ΔPi_ss_, p = 0.002). No significant changes in PCr and Pi kinetics were found during the recovery period after administration of rhuEPO (see figure 3).

### Correlation Analysis

Biochemical analyses of the respiratory chain, MRS measurements as well as frataxin levels [7] showed correlation to patients’ demographical data at baseline. Change in PCr values of the recovery phase correlated significantly between the left and right leg (r = 0.661, p = 0.002). Moreover, ΔPCrss of the recovery period showed an indirect correlation with SARA score (r = −0.832, p<0.001) and number of repeats (r = −0.649; p = 0.016) and therefore direct correlation to age of onset of disease (r = 0.611; p = 0.026). In contrast, neither Pi time constants of the recovery period nor during incremental exercise showed a correlation to demographic and clinical data in FRDA. Frataxin levels measured in skeletal muscle tissue lacked correlation to ΔPCr_ss_ values of the recovery period before (r = 0.453, p = 0.366) and after (r = 0.354, p = 0.491) rhuEPO treatment. On the level of biochemical analyses of the respiratory chain SARA score correlated indirectly with the activity of complex II/III (r = −0.927, p = 0.003) and complex IV (r = −0.927, p = 0.003), whereas complex I and SARA score did not show any correlation (r = −0.360, p = 0.427). Furthermore complex II/III showed a direct correlation with ΔPCr_ss_ values of the recovery period (r = 0.872; p = 0.024) and Pi concentration at termination of exercise (r = 0.853, p = 0.031). Moreover, absolute concentrations of PCr (r = −0.834, p = 0.039) and Pi (r = 0.884, p = 0.019) at exercise abruption correlated with complex IV respiratory chain activity.

## Discussion

In this study we investigated the oxidative energy metabolism of the calf muscles in FRDA patients by 31P MRS using a rest-exercise-recovery protocol. Compared to an age- and sex-matched healthy control group we found lower steady state levels of PCr and increased accumulation of Pi during incremental plantar flexion in FRDA. After maximal exhaustive exercise FRDA patients showed prolonged time constants and decreased steady state of PCr recovery. PCr time constants after exercise reflect mitochondrial ATP production via OXPHOS and are valuable indicators for mitochondrial function [36], [37]. Our results are therefore in line with earlier studies showing deficient ATP production in skeletal muscle of FRDA patients in a regular maintenance state [10] and after ischemic exercise [9]. During incremental work load accumulation of Pi was excessive in FRDA patients, leading to earlier activity induced ischemia and abruption of exercise. Exercise intolerance is a well known feature of mitochondrial myopathies [36], [37], [38]. Moreover, mitochondrial dysfunction detected by MRS is also found in mitochondrial diseases without clinical evidence of myopathy [39], [40]. Histopathological findings in FRDA skeletal muscle reveal a mixed neurogenic/myopathic atrophy pattern with only subtle mitochondrial features [41]. Along with reduced frataxin expression in skeletal muscle tissue [7] our spectroscopy data suggest primary involvement of skeletal muscle in FRDA patients even without pathological hallmarks of mitochondrial myopathy. The extent of mitochondrial dysfunction in skeletal muscle in the present study correlated to steady state levels of PCr during regeneration, whereas no correlation was found to demographic and genetic values. This is in contrast to previous MRS studies in FRDA showing a direct correlation between the maximum rate of PCr recovery and GAA repeats of the shorter *FXN* allele [9], [10]. The lack of correlation in our series is probably due to a different study population including a broader FRDA phenotype and advanced disease stages. Severe gait disturbance and immobilisation are prone, as seen in healthy humans, to result in reduction of mitochondrial density in skeletal muscle [42], [43], [44]. Primary mitochondrial dysfunction in skeletal muscle may therefore be aggravated as a consequence of muscle atrophy in advanced disease stages.

Impaired frataxin expression in FRDA skeletal muscle has been shown only recently [7]. Thus, its impact on oxidative stress markers in muscle has not been investigated until now. Moreover, it has not been explored whether frataxin up-regulation in response to rhuEPO exposure has an impact on mitochondrial function in skeletal muscle. Besides EPO’s well known function as a regulator of red blood cell production, EPO is critically involved in tissue damage, cell apoptosis and metabolic stress [45]. EPO is thereby thought to exert cytoprotective effects by inducing neovascularisation and enhancing trophic activity. Although an average of 45% frataxin up-regulation was found in muscle tissue after rhuEPO exposure in the present FRDA series [7], rhuEPO exposure had no significant impact on respiratory chain activity in muscle specimen [41]. In line with the above-mentioned neuropathological and biochemical findings in muscle tissue, the present MRS measurements in skeletal muscle could not demonstrate benefit in mitochondrial OXPHOS after continuous rhuEPO stimulation of eight weeks.

In principle, oxidative stress in frataxin deficient cells appears to be primarily related to disturbed mitochondrial iron metabolism [5], [46]. Additionally, impaired formation of iron-sulfur clusters (ISC), especially ISC-containing respiratory complexes I, II, and III, in frataxin deficient tissue increases oxidative stress through premature leakage of electrons and the generation of hydroxyl radicals reacting with Fe2+ (Fenton reaction). We found a correlation between impaired respiratory chain complex II and III activity in muscle tissue and PCr recovery, as well as to absolute concentrations of Pi and PCr at exercise abruption. This indicates 31P MRS measurements to be sensitive and valid for monitoring changes in respiratory chain activity, especially for therapeutic agents that improve respiratory chain activity [47].

In analogy to cardiac muscle, 31P MRS of skeletal muscle in FRDA reveals evidence of mitochondrial dysfunction and impaired oxidative phosphorylation despite only subtle mitochondrial features were found in skeletal muscle biopsies. This finding may, at least partially, explain progressive muscle weakness and fatigue in FRDA patients. A relevant impact of rhuEPO stimulation over 2 months on ISC-containing respiratory complexes and ATP was not detectable by 31P MRS.

## Supporting Information

Protocol S1 **Trial protocol.**
(DOC)

Checklist S1 **CONSORT checklist.**
(DOC)

## References

[1] Harding AE (1981) Friedreich's ataxia: a clinical and genetic study of 90 families with an analysis of early diagnostic criteria and intrafamilial clustering of clinical features. Brain 104: 589–620. 7272714 10.1093/brain/104.3.589

[2] Beauchamp M, Labelle H, Duhaime M, Joncas J (1995) Natural history of muscle weakness in Friedreich's Ataxia and its relation to loss of ambulation. Clin Orthop Relat Res: 270–275. 7634585

[3] Sival DA, Pouwels ME, Van Brederode A, Maurits NM, Verschuuren-Bemelmans CC, et al (2011) In children with Friedreich ataxia, muscle and ataxia parameters are associated. Dev Med Child Neurol 53: 529–534. 21574990 10.1111/j.1469-8749.2011.03931.x

[4] Campuzano V, Montermini L, Molto MD, Pianese L, Cossee M, et al (1996) Friedreich's ataxia: autosomal recessive disease caused by an intronic GAA triplet repeat expansion. Science 271: 1423–1427. 8596916 10.1126/science.271.5254.1423

[5] Pandolfo M, Pastore A (2009) The pathogenesis of Friedreich ataxia and the structure and function of frataxin. J Neurol 256 Suppl 19–17. 19283345 10.1007/s00415-009-1003-2

[6] Schmucker S, Puccio H (2010) Understanding the molecular mechanisms of Friedreich's ataxia to develop therapeutic approaches. Hum Mol Genet 19: R103–110. 20413654 10.1093/hmg/ddq165

[7] Nachbauer W, Wanschitz J, Steinkellner H, Eigentler A, Sturm B, et al (2011) Correlation of frataxin content in blood and skeletal muscle endorses frataxin as a biomarker in Friedreich ataxia. Mov Disord 26: 1935–1938. 21692115 10.1002/mds.23789

[8] Wolf C, Boesch S, Metzler B, Weirich-Schwaiger H, Trieb T, et al (2008) Phosphorus-31 two-dimensional chemical shift imaging in the myocardium of patients with late onset of Friedreich ataxia. Mol Imaging Biol 10: 24–29. 18000714 10.1007/s11307-007-0119-y

[9] Vorgerd M, Schols L, Hardt C, Ristow M, Epplen JT, et al (2000) Mitochondrial impairment of human muscle in Friedreich ataxia in vivo. Neuromuscul Disord 10: 430–435. 10899450 10.1016/s0960-8966(00)00108-5

[10] Lodi R, Cooper JM, Bradley JL, Manners D, Styles P, et al (1999) Deficit of in vivo mitochondrial ATP production in patients with Friedreich ataxia. Proc Natl Acad Sci U S A 96: 11492–11495. 10500204 10.1073/pnas.96.20.11492 PMC18061

[11] Lodi R, Hart PE, Rajagopalan B, Taylor DJ, Crilley JG, et al (2001) Antioxidant treatment improves in vivo cardiac and skeletal muscle bioenergetics in patients with Friedreich's ataxia. Ann Neurol 49: 590–596. 11357949

[12] Boesch S, Sturm B, Hering S, Goldenberg H, Poewe W, et al (2007) Friedreich's ataxia: clinical pilot trial with recombinant human erythropoietin. Ann Neurol 62: 521–524. 17702040 10.1002/ana.21177

[13] Sturm B, Stupphann D, Kaun C, Boesch S, Schranzhofer M, et al (2005) Recombinant human erythropoietin: effects on frataxin expression in vitro. Eur J Clin Invest 35: 711–717. 16269021 10.1111/j.1365-2362.2005.01568.x

[14] Acquaviva F, Castaldo I, Filla A, Giacchetti M, Marmolino D, et al (2008) Recombinant human erythropoietin increases frataxin protein expression without increasing mRNA expression. Cerebellum 7: 360–365. 18581197 10.1007/s12311-008-0036-x

[15] Boesch S, Sturm B, Hering S, Scheiber-Mojdehkar B, Steinkellner H, et al (2008) Neurological effects of recombinant human erythropoietin in Friedreich's ataxia: a clinical pilot trial. Mov Disord 23: 1940–1944. 18759345 10.1002/mds.22294

[16] Nachbauer W, Hering S, Seifert M, Steinkellner H, Sturm B, et al.. (2011) Effects of Erythropoietin on Frataxin Levels and Mitochondrial Function in Friedreich Ataxia - a Dose-Response Trial. Cerebellum. 10.1007/s12311-011-0287-9 21597884

[17] Lai N, Dash RK, Nasca MM, Saidel GM, Cabrera ME (2006) Relating pulmonary oxygen uptake to muscle oxygen consumption at exercise onset: in vivo and in silico studies. Eur J Appl Physiol 97: 380–394. 16636861 10.1007/s00421-006-0176-y PMC4124916

[18] Taylor DJ (2000) Clinical utility of muscle MR spectroscopy. Semin Musculoskelet Radiol 4: 481–502. 11371330 10.1055/s-2000-13172

[19] Grassi B (2005) Delayed metabolic activation of oxidative phosphorylation in skeletal muscle at exercise onset. Med Sci Sports Exerc 37: 1567–1573. 16177610 10.1249/01.mss.0000177472.67419.0a

[20] Forbes SC, Slade JM, Meyer RA (2008) Short-term high-intensity interval training improves phosphocreatine recovery kinetics following moderate-intensity exercise in humans. Appl Physiol Nutr Metab 33: 1124–1131. 19088770 10.1139/H08-099

[21] Schmitz-Hubsch T, du Montcel ST, Baliko L, Berciano J, Boesch S, et al (2006) Scale for the assessment and rating of ataxia: development of a new clinical scale. Neurology 66: 1717–1720. 16769946 10.1212/01.wnl.0000219042.60538.92

[22] Burk K, Malzig U, Wolf S, Heck S, Dimitriadis K, et al.. (2009) Comparison of three clinical rating scales in Friedreich ataxia (FRDA). Mov Disord. 10.1002/mds.22660 19562766

[23] Greiner A, Esterhammer R, Messner H, Biebl M, Muhlthaler H, et al (2006) High-energy phosphate metabolism during incremental calf exercise in patients with unilaterally symptomatic peripheral arterial disease measured by phosphor 31 magnetic resonance spectroscopy. J Vasc Surg 43: 978–986. 16678693 10.1016/j.jvs.2006.01.020

[24] Malucelli E, Lodi R, Martinuzzi A, Tonon C, Barbiroli B, et al (2005) Free Mg2+ concentration in the calf muscle of glycogen phosphorylase and phosphofructokinase deficiency patients assessed in different metabolic conditions by 31P MRS. Dyn Med 4: 7. 15938748 10.1186/1476-5918-4-7 PMC1166570

[25] Frouin F, Duteil S, Lesage D, Carlier PG, Herment A, et al (2006) An automated image-processing strategy to analyze dynamic arterial spin labeling perfusion studies. Application to human skeletal muscle under stress. Magn Reson Imaging 24: 941–951. 16916711 10.1016/j.mri.2005.09.012

[26] Quistorff B, Nielsen S, Thomsen C, Jensen KE, Henriksen O (1990) A simple calf muscle ergometer for use in a standard whole-body MR scanner. Magn Reson Med 13: 444–449. 2325544 10.1002/mrm.1910130311

[27] Esterhammer R, Schocke M, Gorny O, Posch L, Messner H, et al (2008) Phosphocreatine kinetics in the calf muscle of patients with bilateral symptomatic peripheral arterial disease during exhaustive incremental exercise. Mol Imaging Biol 10: 30–39. 18000715 10.1007/s11307-007-0118-z

[28] Schocke MF, Esterhammer R, Kammerlander C, Rass A, Kremser C, et al (2004) High-energy phosphate metabolism during incremental calf exercise in humans measured by 31 phosphorus magnetic resonance spectroscopy (31P MRS). Magn Reson Imaging 22: 109–115. 14972400 10.1016/j.mri.2003.07.001

[29] Schocke MF, Esterhammer R, Arnold W, Kammerlander C, Burtscher M, et al (2005) High-energy phosphate metabolism during two bouts of progressive calf exercise in humans measured by phosphorus-31 magnetic resonance spectroscopy. Eur J Appl Physiol 93: 469–479. 15517340 10.1007/s00421-004-1233-z

[30] Kemp GJ, Meyerspeer M, Moser E (2007) Absolute quantification of phosphorus metabolite concentrations in human muscle in vivo by 31P MRS: a quantitative review. NMR Biomed 20: 555–565. 17628042 10.1002/nbm.1192

[31] Taylor DJ, Bore PJ, Styles P, Gadian DG, Radda GK (1983) Bioenergetics of intact human muscle. A 31P nuclear magnetic resonance study. Mol Biol Med 1: 77–94. 6679873

[32] Nevill AM, Jones DA, McIntyre D, Bogdanis GC, Nevill ME (1997) A model for phosphocreatine resynthesis. J Appl Physiol 82: 329–335. 9029234 10.1152/jappl.1997.82.1.329

[33] Schocke MF, Esterhammer R, Ostermann S, Santner W, Gorny O, et al (2006) High-energy phosphate metabolism during calf ergometry in patients with isolated aorto-iliac artery stenoses. Invest Radiol 41: 874–882. 17099426 10.1097/01.rli.0000246148.09129.42

[34] Steinkellner H, Scheiber-Mojdehkar B, Goldenberg H, Sturm B (2010) A high throughput electrochemiluminescence assay for the quantification of frataxin protein levels. Anal Chim Acta 659: 129–132. 20103114 10.1016/j.aca.2009.11.036

[35] Iotti S, Gottardi G, Clementi V, Barbiroli B (2004) The mono-exponential pattern of phosphocreatine recovery after muscle exercise is a particular case of a more complex behaviour. Biochim Biophys Acta 1608: 131–139. 14871490 10.1016/j.bbabio.2003.11.003

[36] Argov Z, Bank WJ, Maris J, Peterson P, Chance B (1987) Bioenergetic heterogeneity of human mitochondrial myopathies: phosphorus magnetic resonance spectroscopy study. Neurology 37: 257–262. 3808305 10.1212/wnl.37.2.257

[37] Taylor DJ, Kemp GJ, Radda GK (1994) Bioenergetics of skeletal muscle in mitochondrial myopathy. J Neurol Sci 127: 198–206. 7707079 10.1016/0022-510x(94)90073-6

[38] Arnold DL, Taylor DJ, Radda GK (1985) Investigation of human mitochondrial myopathies by phosphorus magnetic resonance spectroscopy. Ann Neurol 18: 189–196. 4037759 10.1002/ana.410180205

[39] Barbiroli B, Montagna P, Cortelli P, Iotti S, Lodi R, et al (1995) Defective brain and muscle energy metabolism shown by in vivo 31P magnetic resonance spectroscopy in nonaffected carriers of 11778 mtDNA mutation. Neurology 45: 1364–1369. 7617199 10.1212/wnl.45.7.1364

[40] Lodi R, Taylor DJ, Tabrizi SJ, Kumar S, Sweeney M, et al (1997) In vivo skeletal muscle mitochondrial function in Leber's hereditary optic neuropathy assessed by 31P magnetic resonance spectroscopy. Ann Neurol 42: 573–579. 9382468 10.1002/ana.410420407

[41] Nachbauer W, Boesch S, Reindl M, Eigentler A, Hufler K, et al (2012) Skeletal muscle involvement in friedreich ataxia and potential effects of recombinant human erythropoietin administration on muscle regeneration and neovascularization. J Neuropathol Exp Neurol 71: 708–715. 22805773 10.1097/NEN.0b013e31825fed76

[42] Berg HE, Dudley GA, Hather B, Tesch PA (1993) Work capacity and metabolic and morphologic characteristics of the human quadriceps muscle in response to unloading. Clin Physiol 13: 337–347. 8370234 10.1111/j.1475-097x.1993.tb00334.x

[43] Ferretti G (1997) The effect of prolonged bed rest on maximal instantaneous muscle power and its determinants. Int J Sports Med 18 Suppl 4S287–289. 9391834 10.1055/s-2007-972728

[44] Ferretti G, Antonutto G, Denis C, Hoppeler H, Minetti AE, et al (1997) The interplay of central and peripheral factors in limiting maximal O2 consumption in man after prolonged bed rest. J Physiol 501 (Pt 3): 677–686. 10.1111/j.1469-7793.1997.677bm.x PMC1159468 9218227

[45] Brines M (2010) The therapeutic potential of erythropoiesis-stimulating agents for tissue protection: a tale of two receptors. Blood Purif 29: 86–92. 20093809 10.1159/000245630

[46] Koeppen AH (2011) Friedreich's ataxia: pathology, pathogenesis, and molecular genetics. J Neurol Sci 303: 1–12. 21315377 10.1016/j.jns.2011.01.010 PMC3062632

[47] Schulz JB, Di Prospero NA, Fischbeck K (2009) Clinical experience with high-dose idebenone in Friedreich ataxia. J Neurol 256 Suppl 142–45. 19283350 10.1007/s00415-009-1008-x PMC4277883

