# Supplementary material for: Bioenergetics of the Calf Muscle in Friedreich Ataxia Patients Measured by 31P-MRS Before and After Treatment with Recombinant Human Erythropoietin
Source: PLoS One. 2013 Jul 29;8(7):e69229. doi: 10.1371/journal.pone.0069229 (PMC3726701; doi:10.1371/journal.pone.0069229)
Supplement: Protocol S1 — Trial protocol. (DOC) [file pone.0069229.s001.doc]

**Study Schedule**

**Effects of rhuEPO on stem cell recruitment and metabolism of skeletal muscle in Friedreich’s Ataxia - a Pilot Study**

Ataxia Clinic, Department of Neurology

Medical University of Innsbruck

Institute of Medical Chemistry

Medical University of Vienna


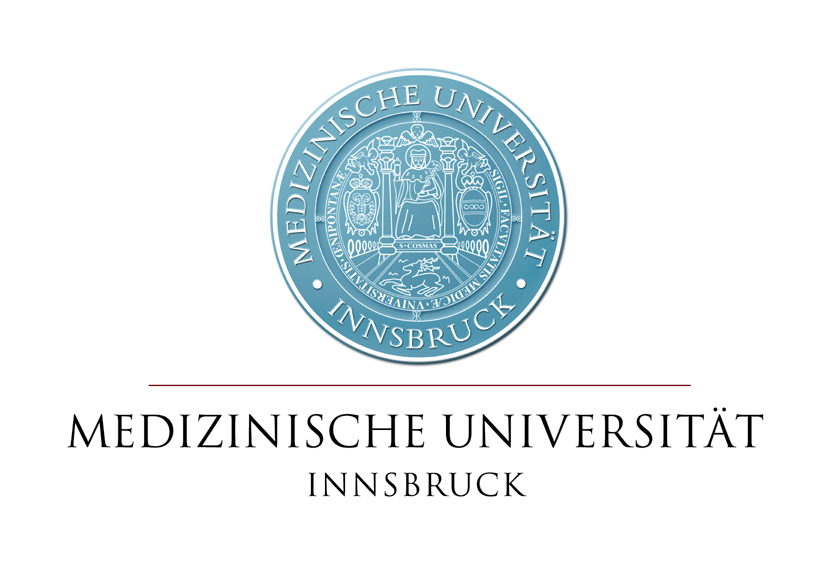


Autoren: Priv. Doz. Dr. Sylvia Boesch, OA Dr. Julia Wanschitz, Prof. Dr. Michael Schocke.

Prof. Dr. Barbara Scheiber-Mojdehkar, o.Prof. Dr. Werner Poewe

Art des Dokuments: Prüfplan

Status des Dokuments: Version 2.0

Phase: II

Datum: 14.02.2008

**Vertraulichkeits-Erklärung**

Dieser Prüfplan unterliegt einschließlich aller seiner Teile dem Urheberrecht. Er ist Eigentum des Studienleiters Dr. Sylvia Boesch, Neurologische Klinik der Medizischen Universität Innsbruck. Dieser Prüfplan darf nicht ohne schriftliche Genehmigung des Studienleiters, Dr. Sylvia Boesch, an Dritte weitergegeben, vervielfältigt oder in elektronischen Systemen weiterverarbeitet werden.

**Scientific background**

**Friedreich Ataxia (FRDA)**

Friedreich ataxia (FRDA) is characterized by slowly progressive ataxia with mean age of onset between age ten and 15 years. The prevalence of FRDA is 2/100,000-4/100,000. The carrier frequency is 1/60-1/100. FRDA is the most common inherited ataxia in Europe, the Middle East, South Asia (Indian subcontinent), and North Africa. FRDA is typically associated with depressed tendon reflexes, dysarthria, muscle weakness, spasticity in the lower limbs, optic nerve atrophy, scoliosis, bladder dysfunction, and loss of position and vibration senses. More than 50% of individuals with FRDA have cardiomyopathy, 30% have diabetes mellitus, and about 25% have an "atypical" presentation with later onset, retained tendon reflexes, or unusually slow progression of disease. Individuals with FRDA have identifiable mutations in the Frataxin (*FXN)* gene. The most common mutation, that accounts for more than 96% of individuals with FRDA, is a GAA triplet-repeat expansion in intron 1 of *FXN* which results in a loss of function type mutation. About 4% of individuals affected with FRDA are compound heterozygotes for a GAA expansion in the disease-causing range in one *FXN* allele and an inactivating *FXN* mutation in the other allele. Disease-causing expanded alleles: 66-1700 GAA repeats. The majority of expanded alleles contain between 600 and 1200 GAA repeats (Campuzano et al 1996, Durr et al 1996, Filla et al 1996, Epplen et al 1997). Frataxin is required for biogenesis of iron-sulfur cluster (ISC), and therefore for the synthesis of enzymes of the respiratory chain complexes I-III and aconitases. Endocardial biopsies of individuals with FRDA revealed a deficiency of ISC proteins (Rotig et al 1997). Frataxin is also thought to play a role in regulation of mitochondrial iron content. Affected individuals show evidence of abnormal accumulation of mitochondrial iron. However, it is likely that mitochondrial iron accumulation may be a secondary manifestation. Frataxin deficiency leads to reduced antioxidant defense, deficient mitochondrial function, and increased oxidative damage. Affected individuals show deficient ATP production and cellular oxygenation in post-exercise skeletal muscle (Lodi et al 1999, Lynch et al 2002) and defective myocardial energy production (Lodi et al 2001, Bunse et al 2003). Penetrance is complete in homozygotes with typical GAA repeat expansions and in compound heterozygotes for an expansion and another deleterious mutation. However, because of wide variability in the size of pathogenic expanded alleles, and for other unknown reasons, onset can range from before age five years to older than age 50 years. Despite the general genotype-phenotype correlations it is not possible to predict the specific clinical outcome in any individual based on genotype. The remaining variability in individuals with FRDA may be caused by genetic background, somatic heterogeneity of the GAA expansion (Montermini, Richter et al 1997; Sharma et al 2002; Sharma et al 2004), and other unidentified factors. The age of onset, presence of leg muscle weakness/wasting, duration until wheelchair use, and prevalence of cardiomyopathy, *pes cavus*, and scoliosis have all shown statistically significant correlations with GAA expansion size (Durr et al 1996; Filla et al 1996; Monros et al 1997; Montermini, Richter et al 1997). The size of the shorter of the two expanded GAA repeats shows better correlation, accounting for about 50% of the variation in age of onset (Filla et al 1996). Cardiomyopathy is more frequently seen with longer GAA repeat alleles (Durr et al 1996, Filla et al 1996, Monros et al 1997). Isnard et al (1997) found echocardiographic evidence of left ventricular hypertrophy in 81% of those with FRDA with repeat lengths greater than 770 triplets and in only 14% of those with repeat lengths less than 770 triplets. Diabetes mellitus or abnormal glucose tolerance does not show a clear-cut correlation with the size of the GAA expansion. Filla et al (1996) found that individuals with diabetes mellitus tend to have larger repeat lengths; in a larger cohort, however, Durr et al (1996) did not find significant correlation either with the size of the GAA expansion or with disease duration. Despite the lack of correlation with the GAA expansion size, Delatycki, Paris et al (1999) found a correlation between the incidence of diabetes mellitus and an earlier age of onset.

Current treatment of FRDA includes symptomatic therapy and devices, there is no medical treatment available. Antioxidant therapy by free radical scavengers such as coenzyme Q10, vitamin E, idebenone (a short-chain analog of coenzyme Q10), and more recently, mitochondrial-targeted idebenone (MitoQ) have been considered potential therapies for slowing the progression of FRDA.

**FRDA and Erythropoietin**

Erythropoetin (EPO) is a 30.400-dalton glycoprotein that was first recognized to regulate red cell production. In humans erythropoetin is produced by peritubular cells in the kidneys of the adult and in hepathocytes in the fetus. The actions of erythropietin in neuroprotection, independent of changes in erythrocyte numbers, are supposed to be due to two mechanisms. First, erythropoietin can act within the *in vivo* context to reverse vasospasm, protects vascular endothelial cells, and stimulates endothelial progenitor cell mobilization (Heeschen et al., Blood 2003) and thus might increase the regenerative capacity of skeletal muscle. EPO modulates inflammation and recruits stem cells. Second, erythropoietin can act directly on neurons by currently unknown mechanisms. Thus, it is supposed to attenuate the production of damaging molecules such as radical oxygen species (ROS) or glutamate-stimulated excitotoxicity.

In several studies on the *in-vitro* and *in-vivo* effects of rhuEPO we were able to detect beneficial effects of rhuEPO in Friedreich’s ataxia. The lack of Frataxin, the disease causing protein deficiency, is partially reversed during rhuEPO treatment, radical oxygen species are significantly reduced in FRDA patients and clinical effects have been found in a recent long term treatment study over six months (personal communication). Since the pathological cascade in FRDA is not exquisite to the central nervous system, but also involves peripheral nerves and skeletal muscle, we intend to investigate circulating and intramuscular endothelial progenitor cells, neovascularization and oxidative metabolism of skeletal muscle in rhuEPO treated FRDA patients.

**Skeletal muscle in FRDA**

Skeletal muscle is a highly specialized postmitotic tissue with a high-level oxidative metabolism. Involvement of skeletal muscle in FRDA has been related to defects of mitochondrial energy metabolism and increased sensitivity to oxidative stress based on sparse morphological studies of muscle tissue of FRDA patients with the findings of mitochondrial proliferation and iron accumulation and deficits of respiratory chain complex activities (Lodi et al. 2006). Additionally, *in-vivo* studies of skeletal and cardiac muscle using magnetic resonance spectroscopy demonstrated impairment of adenosine triphosphate synthesis of skeletal and cardiac muscle in FRDA (Lodi et al. 1999). The spectrum of morphological and biochemical abnormalities of skeletal muscle in a series of genetically confirmed FRDA patients, however, was not systematically investigated.

Maintenance of adult skeletal muscle and regeneration upon damage is largely dependent on satellite cells, a population of tissue-resident committed myogenic progenitors, located in anatomical cell niches beneath the basal lamina of mature myofibers (Mauro 1961) (Schultz and McCormick 1994). After increased work-load or in response to muscle injury, satellite cells enter a well-defined sequence of activation, proliferation, myogenic differentiation and fusion events to repair or replace damaged muscle fibers (Buckingham et al. 2003). The entry of quiescent satellite cells into the myogenic differentiation program is critically regulated by the paired box transcription factors Pax3/Pax7 (Seale et al. 2000) (Relaix et al. 2005) (Zammit et al. 2006) and myogenic regulatory factors including Myf5, MyoD, myogenin and MRF4 (Tajbakhsh and Buckingham 2000) (Kassar-Duchossoy et al. 2004). Additionally sufficient vascularisation is crucial for the integrity and function of skeletal muscle. Increasing evidence by experimental studies suggests recruitment of bone-marrow derived circulating myogenic and endothelial progenitor cells which colonize skeletal muscle via the peripheral blood and contribute to postnatal myo-vasculogenesis. A subpopulation of primitive hematopoietic progenitor cells (HPCs), defined by the surface molecules CD34 and CD133 (Asahara et al. 1997) (Yin et al. 1997) (Gehling et al. 2000), has been found in adults with high potential to differentiate along myo-endothelial lineages *in vitro* (Pesce et al. 2003) (Torrente et al. 2004) and to contribute to regenerating muscle fibers (Jankowski et al. 2002) (Torrente et al. 2004) and neovascularization *in vivo* (Kawamoto et al. 2006). EPO is a potent stimulus for endothelial progenitor cell mobilization (Heeschen et al. 2003) and exhibits high angiogenic potential (Jaquet et al. 2002) (Urao et al. 2006), thus EPO might improve the regenerative capacity and function of skeletal muscle.

**Contributions from the investigators**

**Studies on the Effect of rhuEPO in FRDA**

**Frataxin up-regulation by human recombinant Erythropoietin**

In a first *in vitro* study we showed that recombinant human erythropoietin (rhuEPO) increases frataxin levels in isolated lymphocytes from FRDA patients. Additionally rhuEPO can significantly increase frataxin levels in primary human cardiomyocytes and cardiofibroblasts and neuronal cells (Sturm et al., 2005). Incubation of primary lymphocytes of FRDA patients showed a significant increase of frataxin-levels.

Since there was no animal model available and rhuEPOs long-term use in anaemic patients in the last two decades, an eight weeks “proof-of-concept” study in 10 FRDA patients was perfomed in order to detect a stable in-vivo up-regulation of frataxin in humans. FRDA patients received 5.000 I.U. rhuEPO three times per week subcutaneously for a time period of eight weeks. Frataxin-levels measured by ELISA revealed a stable and significant rise in 7 out of 10 FRDA patients (Boesch et al., 2007). In addition, established markers of oxidative stress such as urine 8-hydroxydeoxyguanosine (a marker for oxidative mitochondrial and nuclear DNA damage) and serum peroxides were significantly reduced after 8 weeks as compared to baseline.

**References:**

Sturm B, Stupphann D, Kaun C, **Boesch S**, Schranzhofer M, Wojta J, Goldenberg H, Scheiber-Mojdehkar B. (*2005)* Recombinant human erythropoietin: effects on frataxin expression in vitro. *Eur J Clin Invest. Nov;35(11):711-7.*

**Boesch S**, Sturm B, Hering S, Goldenberg H, Poewe W, Scheiber-Mojdehkar B. (*2007)* Friedreich's ataxia: clinical pilot trial with recombinant human erythropoietin. *Ann Neurol. Nov;62(5):521-4.*

Sturm B, **Boesch S**, Hering S, Goldenberg H, Poewe W, Scheiber-Mojdehkar B. (2007) Effects of long-term treatment with recombinant human erythropoietin on iron parameters in non-anaemic patients with Friedreich`s ataxia, Brief report, submitted to Blood.

**Boesch S**, Sturm B, Hering S, Scheiber-Mojdehkar B, Steinkellner H, Goldenberg H, Poewe W. (2007) Neurological Effects of Recombinant Human Erythropoietin in Friedreich’s Ataxia: A Clinical Pilot Trial, submitted to Neurology.

**Studies of CD34^+^ and CD133^+^ hematopoietic progenitor cells in human skeletal muscle**

**Evidence for *in vivo* endothelial and myogenic differentiation of CD34^+^ and CD133^+^ hematopoietic progenitor cells in human skeletal muscle**

Using immunohistochemistry and reverse transcriptase-polymerase chain reaction (RT-PCR) we detected mononuclear cells expressing the early hematopoietic progenitor cell (HPC) markers CD34 and CD133 in human skeletal muscle of patients with inflammatory myopathies and normal controls without myopathy, who had a muscle biopsy for the exclusion of malignant hyperthermia. In the context of inflammation numbers of CD34^+^ and CD133^+^ mononuclear cells were significantly increased within the endomysial connective tissue contrasting with low numbers of these cells in controls without myopathy, suggesting that along with the infiltrating myelomonocytic cell population hematopoietic progenitor cells are mobilized from the bone marrow into the circulation and recruited to injured muscle to augment repair mechanisms following inflammatory damage. Several CD34^+^ interstitial cells co-expressed von Willebrand Factor at confocal laser scanning microscopy indicating in-situ maturation of CD34^+^ HPCs to the endothelial lineage. Occasionally, we observed mononuclear cells co-expressing CD133, CD34 and Pax7, a marker of early myogenic differentiation, indicating myogenic commitment of some HPCs.

**References:**

Hollemann D, Budka H, Löscher WN, Yanagida G, Fischer MB, **Wanschitz J.** Endothelial and myogenic differentiation of hematopoietic progenitor cells in inflammatory myopathies, submitted to the Journal of Neuropathology and Experimental Neurology.

**Aim of the project**

The function of frataxin is still unknown, but strong evidence supports the concept that is critically involved in mitochondrial function and oxidative metabolism. Our previous studies demonstrate increase of frataxin-levels in lymphocytes of FRDA patients after rhuEPO administration. However, the pathological cascade in FRDA is not exquisite to the central nervous system, but also involves peripheral nerves and skeletal muscle. We therefore intent to investigate effects of rhEPO on circulating and intramuscular endothelial progenitor cells, neovascularization and oxidative metabolism of skeletal muscle in FRDA.

Numbers of circulating CD34 and CD133 hematopoietic progenitor cells will be measured in the peripheral blood by FACS analysis at baseline and bi-weekly throughout the study phase. Muscle biopsy will be performed at baseline and after two months rhuEPO treatment to study effects of EPO on Frataxin expression, numbers of CD34 and CD133 hematopoietic progenitor cells, satellite cells, capillary density and respiratory chain complex activities.

**The aims of the proposed study are**

1) to investigate morphological and biochemical parameters of skeletal muscle systematically, to identify the amount of CD34^+^ and CD133^+^ hematopoietic progenitor cells as well as to measure the Frataxin expression in skeletal muscle biopsies of FRDA patients at baseline.

2) to measure numbers of circulating CD34^+^ and CD133^+^ hematopoietic progenitor cells in peripheral blood of FRDA patients during treatment with rhuEPO;

3) to study effects of EPO treatment on Frataxin expression, numbers of CD34^+^ and CD133^+^ hematopoietic progenitor cells, satellite cells, capillary density and respiratory chain complex activities of skeletal muscle tissue obtained by re-biopsy.

4) Magnetic resonance spectroscopy in skeletal muscle at baseline and study endpoint will be additionally performed to analyze *in-vivo* changes in muscle energy metabolism.

**Study Design**

This is a single centre, non randomized pilot study.

**Number of patients**

5-7 patients

**Inclusion criteria:**

- Age > 18 years.
- Diagnosis of Friedreich Ataxia (genetic and clinical)
- Einwilligungsfähigkeit muss gegeben sein.
- Informed written consent must be given.

**Exclusion criteria:**

- hematocrit greater than 50%
- thrombocytosis
- cancer
- chronic inflammatory diseases
- unstable diabetes mellitus (HbA1c>8%)
- chronic liver disease
- alcohol abuse
- epilepsy
- heart insufficiency (NYHA>2)
- previous thrombo-embolic events
- anti-coagulation
- pregnancy or breast feeding
- iron, vitamin B12 and/or folic acid deficiency
- cardiovascular diseases
- psychiatric disorders
- hypersensitivity to rhuEPO
- participation in different clinical trials

**Patients and Investigations**

Seven adult subjects with definite FRDA will be enroled in this study. Skeletal muscle will be obtained by an open biopsy from at baseline (untreated) and after 2 months of rhuEPO treatment after written informed consent.

Treatment encompasses rhuEPO 3.000 IU (~100 IU/kg KG) three times weekly subcutaneously for 8 weeks.

Specimen from the quadriceps muscle and MR spectroscopy from the contralateral side will be obtained pre- and post rhuEPO treatment.

Frataxin-levels will be assessed in lymphocytes and muscle specimen at baseline and study end-point. Furthermore, FACS analysis of circulating CD34^+^ and CD133^+^ cells in peripheral blood will be perfomed bi-weekly. The numbers of CD34^+^ and CD133^+^ cells and capillary density per mm^2^ will be quantified in skeletal muscle at baseline and after 8 weeks EPO treatment.

Biochemically, complex I, II, and III of the respiratory chain in the quadriceps muscle will be evaluated pre-and post treatment.

**Synopsis**

| **Assessments and Procedures** | **SC1** | **BL2** | **Visit3** | **Visit4** | **Visit5** | **Visit6** | **FU7** |
| --- | --- | --- | --- | --- | --- | --- | --- |
| **Timepoint (weeks)** |  | **0** | **2** | **4** | **6** | **8** | **10-15** |
| Informed Consent | **X** |  |  |  |  |  |  |
| Medical History | **X** |  |  |  |  |  |  |
| Physical Examination | **X** |  |  |  |  | **X** | **X** |
| Vital Signs | **X** | **X** |  | **X** |  | **X** | **X** |
| **Muscle Biopsy** |  | **X** |  |  |  | **X** |  |
| Muscle MRS |  | **X** |  |  |  | **X** |  |
| FACS | **X** | **X** |  | **X** |  | **X** | **X** |
| Clinical Lab. Tests | **X** | **X** | **X** | **X** | **X** | **X** | **X** |
| Frataxin |  | **X** |  |  |  | **X** |  |
| **SARA** | **X** | **X** |  | **X** |  | **X** | **X** |
| PRO |  | **X** |  |  |  | **X** | **X** |
| AE Assessment |  | **X** | **X** | **X** | **X** | **X** | **X** |
| Concomitant Meds. | **X** | **X** | **X** | **X** | **X** | **X** | **X** |

Abbreviations: SC, screening; BL, baseline; FU, follow-up visit; MRS, magentic resonance spectroscopy ; SARA, Scale for the Assessment and Rating of Ataxia, PRO, patient-reported outcome measures; AE, adverse event

**Methods**

After muscle biopsy the tissue will immediately be snap frozen in liquid nitrogen and stored at -70°C until analysis.

**Enzymehistochemistry and Immunohistochemistry**

Frozen sections of vastus lateralis muscle will be studied by standard histological procedures. Immunohistochemistry for endothelial and myogenic markers will be performed as previously reported (Ruger et al. 2004) with minor modifications. Acetone fixed sections will be blocked with avidin/biotin blocking reagent (Vector Laboratories, Burlingame, CA) and incubated overnight at 4°C with a rabbit anti-von Willebrand factor (vWF, 5.7 µg/ml, Dako), mouse anti-CD31 (4.5 µg/ml, Dako), mouse anti-CD34 (0.5 µg/ml, Beckman Coulter, Immunotech, Marseille, France), mouse anti-CD133 (5 µg/ml, R&D Systems, Minneapolis, MN, detecting AC133-1 and AC133-2), and mouse anti-Pax7 (7.1 µg/ml, R&D Systems) diluted in Tris buffered saline (TBS)/1% bovine serum albumin (BSA). The reactivity of the primary unlabelled antibodies will be revealed using biotinylated goat F(ab’)_2_ anti-mouse IgG+IgM (H+L) (4.2 µg/ml, Jackson ImmunoResearch, West Grove, PA) or biotinylated donkey anti-rabbit IgG (H+L) (1.2 µg/ml, Jackson ImmunoResearch) diluted in 500 µg/ml normal human Ig (Biotest, Dreieich, Germany) followed by streptavidin-horseradish peroxidase (HRP) complex (20 µg/ml, Sigma, St. Louis, MO). For the detection of CD133 a supersensitive streptavidin-HRP conjugate (Biocare medical, Walnut Creek, CA) will be used. To visualize antibody staining the sections will be exposed to 3-amino-9-ethyl-carbazole (AEC, Sigma), and subsequently counterstained with Mayer’s hemalaun.

**Confokal Laser Scanning Microscopy**

4 µm thick frozen sections will be acetone fixed, blocked with avidin/biotin blocking reagent and 12.5% donkey serum, incubated overnight at 4°C with mouse anti-Pax7 or rabbit anti-vWF diluted in TBS/1% BSA, and subsequently incubated with 500 µg/ml normal human Ig and Alexa Fluor (AF) 555 donkey anti-mouse IgG (H+L) (for mouse anti-Pax7, all secondary AF antibodies 4 µg/ml, Invitrogen Life Technologies) or AF 555 donkey anti-rabbit IgG (H+L) (for rabbit anti-vWF). After blocking with 20% mouse serum (for anti-Pax7), tissue samples will then be incubated with fluorescein isothiocyanate (FITC)-conjugated anti-CD34 (2.5 µg/ml, Becton Dickinson PharMingen, San José, CA) diluted in TBS/1% BSA/5% mouse serum overnight at 4°C. An incubation with polyclonal goat anti-CD133 (2 µg/ml, Santa Cruz Biotechnology) overnight will be followed by a step using secondary antibody AF 647 donkey anti-goat IgG (H+L) (4 µg/ml, Invitrogen) which finishes the staining procedure. Stained samples will be covered with 4’, 6-diamidino-2-phenylindole (DAPI), diluted 1:4 with Vectashield mounting medium (Vector Laboratories). Sections will be analyzed with a confocal laser-scanning microscope (Zeiss LSM 510, Oberkochen, Germany) with a multiphoton laser (argon laser: 488 nm for FITC (green); Helium Neon (HeNe) 1: 543 nm for AF 555 (red); HeNe 2: 633 nm for AF 647 (blue)) and a 63 × Zeiss Plan-Apochromat differential interference contrast oil immersion objective with numerical aperture 1.40 using multitracking scan (Ruger et al. 2004).

**Morphometric analysis**

To quantify CD34^+^ and CD133^+^ progenitor cells, Pax7^+^ satellite cells, and CD31^+^ and von Willebrand factor^+^ mature endothelial cells 16 representative areas per section within the endomysium will be selected. Numbers of cells will be counted with a 40x objective using an ocular morphometric grid covering a total area of 1 mm^2^ (Wanschitz et al. 2003).

**Biochemistry**

Biochemical analysis of complexes I-IV will be performed from immediately frozen muscle tissue according to standard procedures.

**FACS analysis**

FACS analysis of CD34^+^ and CD133^+^ circulating hematopoietic progenitor cells will be performed twice a week from peripheral blood.

**Phosphor 31 Magnetic Resonance Spectrosopy**

All FRDA patients are subjected to phosphor 31 magnetic resonance spectrosopy (31P MRS) of the left leg during an incremental exercise protocol at 2, 4 and 6 W. By measuring the PCr kinetics and calculating the time constants, mitochondrial function can be quantitated.

# **31P MRS Protocol**

The spectroscopic measurements are performed on a 1.5 Tesla whole body MR scanner (Magnetom Magnetom Avanto, Siemens Erlangen, Germany) by using a circular polarized double resonator surface coil that permits the receipt of 1H resonances at 63.5 MHz and 31P resonances at 25.8 MHz. The transmitter coil has a diameter of 21 cm, the receiver coil has a diameter of 14 cm. A free induction decay (FID) sequence with following parameters is employed: repetition time (TR), 1000 ms; echo time (TE), 0.13 ms; flip angle, 90°; 10 averages; acquisition time (TA), 10 s. The nuclear Overhauser enhancement (NOE) is applied to all spectroscopic measurements. The signal is received from the calf that is fixed on the double resonator coil. The other leg is embedded above the depth range of this coil.

### 31P MRS Data Analysis

The spectral data are processed using the commercial software package as provided by the manufacturer (Siemens Erlangen, Germany). The peak areas of PCr are fitted in the frequency domain. In addition, the position of the peaks of PCr and Pi are determined. Since absolute concentrations are not calculated, the integrals of the metabolite peaks are not corrected for partial saturation and the NOE effects. Moreover, we assume that the T1 relaxation times of the metabolites are constant throughout each 31P MRS session [30;31]. The total extent of PCr hydrolysis is determined by normalization of the end-exercise integral to the baseline integral at rest. The intracellular pH is calculated from the chemical shift of Pi based on the equation: [Taylor et al., Mol.Biol.Med. 1983]

pH=6.75 + log(δ-3.27)/(5.69-δ) (1)

δ is the chemical shift of the Pi peak in parts per million (ppm) relative to PCr.

The time constants τ and ΔPCr_ss_ values, meaning the difference between baseline and steady-state PCr level are calculated for each workload increment by using a non-linear regression analysis in SPSS 15.0 for windows (SPSS Inc., Chicago, Illinois, U.S.A.), as previously reported [Nevill et al., JAP 1997]. For that purpose, the last PCr integral of the preceding increment or rest phase is considered baseline PCr integral for the following increment and for data normalization. The time constants τ and ΔPCr_ss_ values are iteratively calculated by using the equation: [Schocke Invest Radiol 2006, Greiner JVS 2006, Esterhammer Mol.Imaging.Biol. in press]

ΔPCr(*_t_*) = PCr_0_ -/+ ΔPCr_ss_ [1-*e^-t^*^/τ^] (2)

PCr_0_ is the baseline value, ΔPCr(*_t_*) the PCr value at the time (*t*) and ΔPCr_ss_ the difference between baseline PCr value and the estimated steady-state level. The coherence between the mono-exponential model and the data of each subject is described by a coefficient of determination r^2^: [Schocke Invest Radiol 2006, Greiner JVS 2006, Esterhammer Mol.Imaging.Biol. in press]

r^2^ = 1 - residual sum of squares / corrected sum of squares (3)

A coefficient r^2^ above 0.4 is considered an acceptable fit, whereas a coefficient r^2^ below 0.4 indicated a weak agreement with a monoexponential model and rather progressive PCr breakdown.

**Reference:**

Wolf C, **Boesch S**, Metzler B, Weirich-Schwaiger H, Trieb T, Schocke MF.Phosphorus-31 Two-Dimensional Chemical Shift Imaging in the Myocardium of Patients with Late Onset of Friedreich Ataxia. Mol Imaging Biol. 2007 Nov 14;

**Statistical analysis**

Depending on the distribution of variables (analyzed by the Kolmogorov-Smirnov test) parametric (i.e. one-way analysis of variance (ANOVA); Pearson’s correlation coefficient for univariate correlation analyses) or non-parametric tests (Kruskal-Wallis test; Mann-Whitney-U test for group comparisons, Spearman’s correlation coefficient for univariate correlation analyses) will be used. Multivariable linear regression analysis, controlling for possible confounding covariates, will be performed for the variables found to be significant in the univariate models. Categorial variables will be analysed by chi-square test and logistic regression analysis.

**Interventions**

**Treatment**

Human recombinant erythropoietin (rhuErypo) will be applyed three times weekly. The dosage will be 3.00 IU Neo-Recormon (Roche). The appication of the medication will be done by the patient in the gluteal area: In case of iron deficiency (anemia) Fe will be substituted. (z.B. 200 mg elementares Eisen/Tag).

**Hemoglobin/Modification of dosage**

In case of increase in Hämoglobin values >185 g/l in male patients (Grenzwert 130-177 g/l) und >165 g/l (Grenzwert 120-157 g/l) in female patients Phlebotomy will be performed (150 -200 ml).

**Phlebotomy**

**Hb >185 g/l in males (Grenzwert 130-177 g/l)**

**Hb >165 g/l in females (Grenzwert 120-157 g/l)**

**>>>> Phlebotomy 150-200 ml Blood <<<<**

**References**

Asahara T, Murohara T, Sullivan A et al. Isolation of putative progenitor endothelial cells for angiogenesis. Science. 1997; 275(5302): 964-7

Boesch S, Sturm B, Hering S, Scheiber-Mojdehkar B, Steinkellner H, Goldenberg H, Poewe W. (2007) Neurological Effects of Recombinant Human Erythropoietin in Friedreich’s Ataxia: A Clinical Pilot Trial, submitted to Neurology.

Boesch S, Sturm B, Hering S, Goldenberg H, Poewe W, Scheiber-Mojdehkar B. (*2007)* Friedreich's ataxia: clinical pilot trial with recombinant human erythropoietin. *Ann Neurol. Nov;62(5):521-4.*

Bogoyevitch MA. An update on the cardiac effects of erythropoietin cardioprotection by erythropoietin and the lessons learnt from studies in neuroprotection. Cardiovasc Res. 2004; 63:208-216

Brines ML, Ghezzi P, Keenan S et al. Erythropoietin crosses the blood-brain barrier to protect against experimental brain injury. Proc Natl Acad Sci U S A. 2000; 97:10526-10531

Buckingham M, Bajard L, Chang T et al. The formation of skeletal muscle: from somite to limb. J Anat 2003; 202(1): 59-68

Bunse M, Bit-Avragim N, Riefflin A, Perrot A, Schmidt O, Kreuz FR, Dietz R, Jung WI, Osterziel KJ (2003) Cardiac energetics correlates to myocardial hypertrophy in Friedreich's ataxia. *Ann Neurol* 53:121-3

Calapai G, Marciano MC, Corica F et al. Erythropoietin protects against brain ischemic injury by inhibition of nitric oxide formation. Eur J Pharmacol. 2000; 401:349-356

Campuzano V, Montermini L, Lutz Y et al. Frataxin is reduced in Friedreich ataxia patients and is associated with mitochondrial membranes. Hum Mol Genet. 1997; 6:1771-1780

Campuzano V, Montermini L, Lutz Y, Cova L, Hindelang C, Jiralerspong S, Trottier Y, Kish SJ, Faucheux B, Trouillas P, Authier FJ, Durr A, Mandel JL, Vescovi A, Pandolfo M, Koenig M (1997) Frataxin is reduced in Friedreich ataxia patients and is associated with mitochondrial membranes. *Hum Mol Genet* 6:1771-80

Campuzano V, Montermini L, Molto MD et al. Friedreich's ataxia: autosomal recessive disease caused by an intronic GAA triplet repeat expansion. Science. 1996; 271:1423-1427

Campuzano V, Montermini L, Molto MD, Pianese L, Cossee M, Cavalcanti F, Monros E, Rodius F, Duclos F, Monticelli A, et al (1996) Friedreich's ataxia: autosomal recessive disease caused by an intronic GAA triplet repeat expansion. *Science* 271:1423-7

Chong ZZ, Kang JQ, Maiese K. Erythropoietin is a novel vascular protectant through activation of Akt1 and mitochondrial modulation of cysteine proteases. Circulation. 2002; 106:2973-2979

Delatycki MB, Paris DB, Gardner RJ, Nicholson GA, Nassif N, Storey E, MacMillan JC, Collins V, Williamson R, Forrest SM (1999) Clinical and genetic study of Friedreich ataxia in an Australian population. *Am J Med Genet* 87:168-74

Digicaylioglu M, Lipton SA. Erythropoietin-mediated neuroprotection involves cross-talk between Jak2 and NF-kappaB signalling cascades. Nature. 2001; 412:641-647

Durr A, Cossee M, Agid Y, Campuzano V, Mignard C, Penet C, Mandel JL, Brice A, Koenig M (1996) Clinical and genetic abnormalities in patients with Friedreich's ataxia. *N Engl J Med* 335:1169-75

Epplen C, Epplen JT, Frank G, Miterski B, Santos EJ, Schols L (1997) Differential stability of the (GAA)n tract in the Friedreich ataxia (STM7) gene. *Hum Genet* 99:834-6

Filla A, De Michele G, Cavalcanti F, Pianese L, Monticelli A, Campanella G, Cocozza S (1996) The relationship between trinucleotide (GAA) repeat length and clinical features in Friedreich ataxia. *Am J Hum Genet* 59:554-60

Gehling UM, Ergun S, Schumacher U et al. In vitro differentiation of endothelial cells from AC133-positive progenitor cells. Blood 2000; 95(10): 3106-12

Grasso G, Buemi M, Alafaci C et al. Beneficial effects of systemic administration of recombinant human erythropoietin in rabbits subjected to subarachnoid hemorrhage. Proc Natl Acad Sci U S A. 2002; 99:5627-5631

Harding AE. Friedreich's ataxia: a clinical and genetic study of 90 families with an analysis of early diagnostic criteria and intrafamilial clustering of clinical features. Brain. 1981; 104:589-620

Heeschen C, Aicher A, Lehmann R et al. Erythropoietin is a potent physiologic stimulus for endothelial progenitor cell mobilization. Blood 2003; 102(4): 1340-6

Isnard R, Kalotka H, Durr A, Cossee M, Schmitt M, Pousset F, Thomas D, Brice A, Koenig M, Komajda M (1997) Correlation between left ventricular hypertrophy and GAA trinucleotide repeat length in Friedreich's ataxia. *Circulation* 95:2247-9

Jankowski RJ, Deasy BM, Cao B et al. The role of CD34 expression and cellular fusion in the regeneration capacity of myogenic progenitor cells. J Cell Sci 2002; 115(Pt 22): 4361-74

Jaquet K, Krause K, Tawakol-Khodai M et al. Erythropoietin and VEGF exhibit equal angiogenic potential. Microvasc Res 2002; 64(2): 326-33

Juul S. Erythropoietin in the central nervous system, and its use to prevent hypoxic-ischemic brain damage. Acta Paediatr Suppl. 2002; 91:36-42

Kassar-Duchossoy L, Gayraud-Morel B, GomesD et al. Mrf4 determines skeletal muscle identity in Myf5:Myod double-mutant mice. Nature 2004; 431(7007): 466-71

Kawakami M, Sekiguchi M, Sato K et al. Erythropoietin receptor-mediated inhibition of exocytotic glutamate release confers neuroprotection during chemical ischemia. J Biol Chem. 2001; 276:39469-39475

Kawamoto A, Iwasaki H, Kusano K et al. CD34-positive cells exhibit increased potency and safety for therapeutic neovascularization after myocardial infarction compared with total mononuclear cells. Circulation 2006; 114(20): 2163-9

Li W, Maeda Y, Yuan RR et al. Beneficial effect of erythropoietin on experimental allergic encephalomyelitis. Ann Neurol. 2004; 56:767-777

Lodi R, Cooper JM, Bradley JL et al. Deficit of in vivo mitochondrial ATP production in patients with Friedreich ataxia. Proc Natl Acad Sci U S A 1999; 96(20): 11492-5

Lodi R, Tonon C, Calabrese V et al. Friedreich's ataxia: from disease mechanisms to therapeutic interventions. Antioxid Redox Signal 2006; 8(3-4): 438-43

Lynch DR, Farmer JM, Balcer LJ, Wilson RB (2002) Friedreich ataxia: effects of genetic understanding on clinical evaluation and therapy. *Arch Neurol* 59:743-7

Masuda S, Nagao M, Takahata K et al. Functional erythropoietin receptor of the cells with neural characteristics. Comparison with receptor properties of erythroid cells. J Biol Chem. 1993; 268:11208-11216

Mateo I, Llorca J, Volpini V et al. Expanded GAA repeats and clinical variation in Friedreich's ataxia. Acta Neurol Scand. 2004; 109:75-78

Mauro A. Satellite cell of skeletal muscle fibers. J Biophys Biochem Cytol 1961; 9: 493-5

Monros E, Molto MD, Martinez F, Canizares J, Blanca J, Vilchez JJ, Prieto F, de Frutos R, Palau F (1997) Phenotype correlation and intergenerational dynamics of the Friedreich ataxia GAA trinucleotide repeat. *Am J Hum Genet* 61:101-10

Pesce M, Orlandi A, Iachininoto MG et al. Myoendothelial differentiation of human umbilical cord blood-derived stem cells in ischemic limb tissues. Circ Res 2003; 93(5): e51-62

Relaix F, Rocancourt D, Mansouri A et al. A Pax3/Pax7-dependent population of skeletal muscle progenitor cells. Nature 2005; 435(7044): 948-53

Rotig A, de Lonlay P, Chretien D, Foury F, Koenig M, Sidi D, Munnich A, Rustin P (1997) Aconitase and mitochondrial iron-sulphur protein deficiency in Friedreich ataxia. *Nat Genet* 17:215-7

Ruger B, Giurea A, Wanivenhaus AH et al. Endothelial precursor cells in the synovial tissue of patients with rheumatoid arthritis and osteoarthritis. Arthritis Rheum 2004; 50(7): 2157-66

Schultz E and McCormick KM. "Skeletal muscle satellite cells." Rev Physiol Biochem Pharmacol 1994; 123: 213-57

Schulz JB, Dehmer T, Schols L et al. Oxidative stress in patients with Friedreich ataxia. Neurology. 2000; 55:1719-1721

Seale P, Sabourin LA, Girgis-Gabardo A et al. Pax7 is required for the specification of myogenic satellite cells." Cell 2000; 102(6): 777-86

Shingo T, Sorokan ST, Shimazaki T et al. Erythropoietin regulates the in vitro and in vivo production of neuronal progenitors by mammalian forebrain neural stem cells. J Neurosci. 2001; 21:9733-9743

Siren AL, Ehrenreich H. Erythropoietin--a novel concept for neuroprotection. Eur Arch Psychiatry Clin Neurosci. 2001; 251:179-184

Smith KJ, Bleyer AJ, Little WC et al. The cardiovascular effects of erythropoietin. Cardiovasc Res. 2003; 59:538-548

Squadrito F, Altavilla D, Squadrito G et al. Recombinant human erythropoietin inhibits iNOS activity and reverts vascular dysfunction in splanchnic artery occlusion shock. Br J Pharmacol. 1999; 127:482-488

Sturm B, Boesch S, Hering S, Goldenberg H, Poewe W, Scheiber-Mojdehkar B. (2007) Effects of long-term treatment with recombinant human erythropoietin on iron parameters in non-anaemic patients with Friedreich`s ataxia, Brief report, submitted to Blood.

Sturm B, Stupphann D, Kaun C, Boesch S, Schranzhofer M, Wojta J, Goldenberg H, Scheiber-Mojdehkar B. (*2005)* Recombinant human erythropoietin: effects on frataxin expression in vitro. *Eur J Clin Invest. Nov;35(11):711-7.*

Tajbakhsh S and Buckingham M. The birth of muscle progenitor cells in the mouse: spatiotemporal considerations. Curr Top Dev Biol 2000; 48: 225-68

Tan G, Chen LS, Lonnerdal B et al. Frataxin expression rescues mitochondrial dysfunctions in FRDA cells. Hum Mol Genet. 2001; 10:2099-2107

Torrente Y, Belicchi M, Sampaolesi M et al. Human circulating AC133(+) stem cells restore dystrophin expression and ameliorate function in dystrophic skeletal muscle." J Clin Invest 2004 114(2): 182-95

Urao N, Okigaki M, Yamada H et al. Erythropoietin-mobilized endothelial progenitors enhance reendothelialization via Akt-endothelial nitric oxide synthase activation and prevent neointimal hyperplasia. Circ Res 2006; 98(11): 1405-13

Wanschitz J, Maier H, Lassmann H et al. Distinct time pattern of complement activation and cytotoxic T cell response in Guillain-Barre syndrome. Brain 2003; 126(Pt 9): 2034-42

Wolf C, Boesch S, Metzler B, Weirich-Schwaiger H, Trieb T, Schocke MF. *(2007)* Phosphorus-31 Two-Dimensional Chemical Shift Imaging in the Myocardium of Patients with Late Onset of Friedreich Ataxia. *Mol Imaging Biol. Nov 14;*

Yin AH, Miraglia S, Zanjani ED et al. AC133, a novel marker for human hematopoietic stem and progenitor cells." Blood 1997; 90(12): 5002-12

Zammit PS, Relaix F, Nagata Y et al. Pax7 and myogenic progression in skeletal muscle satellite cells." J Cell Sci 2006; 119(Pt 9): 1824-32
